# Cardiac arrest associated with sildenafil ingestion in a patient with an abnormal origin of the left coronary artery: case report

**DOI:** 10.1186/1471-2261-11-49

**Published:** 2011-08-08

**Authors:** Bruno C Huber, Franz von Ziegler, Fabian Bamberg, Wolfgang-Michael Franz, Alexander Becker

**Affiliations:** 1 Department of Internal Medicine I, Ludwig-Maximilians-University, Campus Grosshadern, Munich, Germany; 2 Department of Radiology, Ludwig-Maximilians-University, Campus Grosshadern, Munich, Germany

## Abstract

**Background:**

Left coronary artery arising from the right sinus of Valsalva is an uncommon congenital coro-nary anomaly that seems to be associated with sudden death in young patients.

**Case presentation:**

We report a case of cardiac arrest in a 59-year-old patient after sexual intercourse and Silde-nafil ingestion. A coronary arteriography and an angiographic computed tomography scan subsequently revealed a LCA origin from the right aortic sinus along with an intramural course of the left main stem. In addition a distal stenosis of the right coronary artery was detected. After successful resuscitation without neurological deficits coronary artery bypass surgery was performed.

**Conclusion:**

To our knowledge, this is the first report demonstrating sudden cardiac arrest associated with Sildenafil ingestion in a patient with this type of coronary anomaly. The question arises, whether a cardiac screening is necessary before a Sildenafil therapy is initiated.

## Background

Coronary artery anomalies (CAAs) are a group of congenital disorders with different manifestations and supposed pathophysiological mechanisms [1]. In literature the overall incidence of CAAs in the general population has been described between 0.3% and 5.64%, which can be explained by the examined inhomogeneous populations and different definitions of CAAs [2]. In case of CAA the interarterial course between the aorta and pulmonary artery is associated with a severe prognosis [1]. In autopsy materials from previously healthy athletes who suffered a sudden death, a right coronary arterial origin from the left coronary sinus and a LCA origin from the right sinus have been described as the most common congenital coronary mal-formations [3]. Sudden death has been sometimes the first manifestation of these abnormalities, whereas CAAs rarely present with symptoms such as angina pectoris, dyspnoe or cardiac arrhythmias. In contrast to CAA with an interarterial course CAA with an extraarterial course are not associated with an increased risk of sudden cardiac death and do not require coronary interventions [1]. We report a case of cardiac arrest associated with Sildenafil ingestion in a patient with a CAA.

## Case presentation

**Figure 1:**
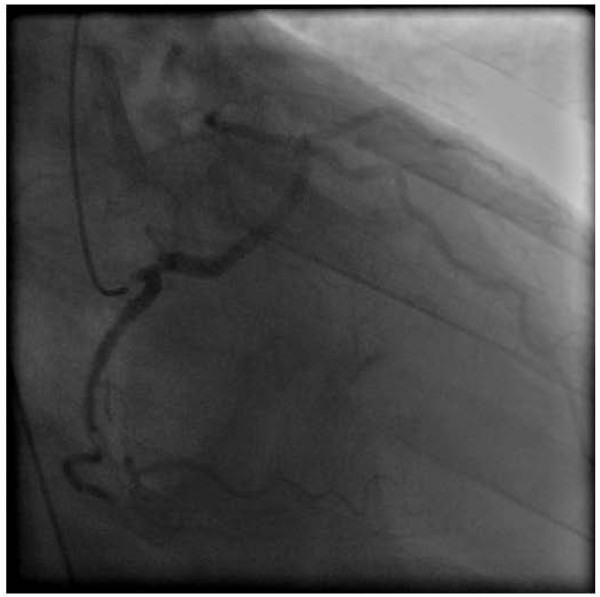
**Coronary arteriography showing an atypical origin of the left coronary artery from the right aortic sinus**.

**Figure 2:**
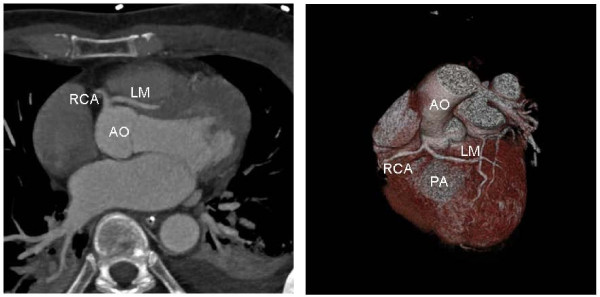
**ECG-gated 64-slice angiographic CT scan, axial image (A) and volume rendered image (B) showing the origin and course of the LM in relation to aorta (AO) and the pulmonary artery (PA)**.

A 59-year-old caucasian man was found by his wife collapsed in the bathroom after sexual intercourse. On arrival the rescue team found an unconscious patient with ventricular fibrillation. After single defibrillation and 15 minutes of cardiopulmonary resuscitation spontaneous circulation was restored. His wife reported that the patient has ingested Sildenafil (50 mg) 30 minutes before the cardiac arrest for the first time. Except from a vascular-type erectile dysfunction he had no previous medical history and no familial history of cardiovascular disease or sudden death. The patient did not take a regular medication. After admission in our hospital a 12-lead electrocardiogram showed no signs of significant repolarization abnormalities. There were increases in cardiac enzymes with a troponin I (TNI) of 5.57 ng/ml (reference range < 0.05 ng/ml), a creatinine-kinase (CK) of 3285 U/l (reference range < 180 U/l), a creatine kinase MB (CKMB) of 124 U/l (reference range < 12 U/l) and a CRP of 2.2 mg/dl (reference range < 0.5 mg/dl). 12 hours later TNI and CKMB had decreased to 2.34 U/l and 87 U/l and at 24 hours they had further decreased to 2.09 U/l and 78 U/l, respectively. Echocardiogram showed impaired left ventricular function, ejection fraction was 46 percent. A coronary arteriography (Figure 1) and an angiographic CT scan (Figure 2) were performed. The coronary arteriography revealed an atypical origin of the left coronary artery from the right aortic sinus as well as a distal stenosis of the right coronary artery. Coronary anomaly was confirmed by the angiographic CT scan which showed the interarterial course of the left coronary artery between the pulmonary trunk and the aorta. Due to these findings he was transferred to the cardiac surgery department and coronary artery bypass surgery was performed with the left mammary artery supplying the left anterior descending artery and two venous grafts supplying the ramus circumflexus and right coronary artery. A proximal ligation of the left anterior descending artery was not performed. After 6 months ventricular function had recovered, echocardiography showed an ejection fraction of 54 percent.

CAAs are a group of congenital disorders which rarely present with symptoms such as angina pectoris, dyspnoe or cardiac arrhythmias. Also in our patient the cardiac arrest was the first event and no other symptoms in the past have been observed. Before the event occurred the patient had ingested Sildenafil (50 mg), an oral phosphodiesterase type 5 (PDE5) inhibitor used for the treatment of erectile dysfunction. It has been demonstrated in several studies that the use of sildenafil is not associated with an increased risk for cardiovascular events in a cardiovascular healthy population [4]. However, Patients with known coronary artery disease using PDE5 inhinbitors are at risk of developing myocardial infarction after receiving sildenafil in combination with nitrates or calcium antagonists due to prolonged and exaggerated vasodilatation and hypotension [5]. In the present case, the combination of sildenafil-triggered vasodilatation and exercise maybe caused a coronary steal phenomenon following ischemia with lethal arrhythmias. Patients with coronary anomalies usually become symptomatic in their childhood or adolescence. The relatively late age of our patient might indicate that sildenafil as a trigger was necessary to induce a perfusion deficit. Another pathogenetic link in exercise related death in patients with CAAs is suggested to be the compression of the intraarterial coronary artery leading to ischemia in these patients [1]. Due to the high risk of sudden cardiac death in patients with an interarterial course of the aberrant coronary artery reinsertation of the coronary ostia is recommended. In case of additional coronary stenoses bypass surgery should be performed.

In patients with an extraarterial course of the aberrant coronary artery an increased risk of sudden cardiac death could not be observed, coronary interventions are not indicated.

## Conclusions

In summary, this case demonstrates that exercise and drugs affecting coronary resistance like Sildenafil might cause ischemia and sudden death in patients with "malignant" coronary artery anomalies and arises the question whether a cardiac screening e.g. by echocardiography might be performed in patients before a Sildenafil therapy is initiated. Of course a link between Sildenafil ingestion and cardiac arrest cannot be conclusively established and a co-occurence of both incidences is possible.

## Consent

Written informed consent was obtained from the patient for publication of this case report and any accompanying images. A copy of the written consent is available for review by the Editor-in-Chief of this journal

## List of abbreviations

CAAs: Coronary artery anomalies; CK: creatinine-kinase; LCA: Left coronary artery; PDE5: phosphodiesterase type 5; TNI: troponin I.

## Competing interests

The authors declare that they have no competing interests.

## Authors' contributions

BCH analyzed and interpreted patient data and was a major contributor in writing the manuscript, FZ and FB carried out and interpreted the CT studies, WMF contributed to interpretation of the studies, AB has been involved in drafting the manuscript and revising it critically for im-portant intellectual content. All authors read and approved the manuscript.

## Pre-publication history

The pre-publication history for this paper can be accessed here:

http://www.biomedcentral.com/1471-2261/11/49/prepub

## References

[B1] Angelini P Coronary artery anomalies: an entity in search of an identity Circulation 2007 115 10 1296 1305 17353457 10.1161/CIRCULATIONAHA.106.618082

[B2] Angelini P Velasco JA Flamm S Coronary anomalies: incidence, pathophysiology, and clinical relevance Circulation 2002 105 20 2449 2454 10.1161/01.CIR.0000016175.49835.57 12021235

[B3] Basso C Maron BJ Corrado D Thiene G Clinical profile of congenital coronary artery anomalies with origin from the wrong aortic sinus leading to sudden death in young competitive athletes J Am Coll Cardiol 2000 35 6 1493 1501 10.1016/S0735-1097(00)00566-0 10807452

[B4] Brindis RG Kloner RA Sildenafil in patients with cardiovascular disease Am J Cardiol 2003 92 9A 26M 36M 14609621 10.1016/s0002-9149(02)03368-4

[B5] Webb DJ Freestone S Allen MJ Muirhead GJ Sildenafil citrate and blood-pressure-lowering drugs: results of drug interaction studies with an organic nitrate and a calcium antagonist Am J Cardiol 1999 83 suppl 5A 21C 28C 10078539 10.1016/s0002-9149(99)00044-2

